# Using scene proximity judgments to study food-specific recognition ability

**DOI:** 10.3758/s13423-025-02845-9

**Published:** 2026-02-17

**Authors:** Conor J. R. Smithson, Yiming Lin, Isabel Gauthier

**Affiliations:** Department of Psychological Sciences, Vanderbilt University, Nashville, TN USA

**Keywords:** Food neophobia, Object recognition, Scene recognition, Individual differences

## Abstract

The visual recognition of food depends on both domain-general and domain-specific visual mechanisms. Food-recognition ability negatively correlates with food neophobia (the tendency to avoid novel foods), possibly because the avoidance of novel food may limit perceptual experience that supports food recognition abilities. In prior work this relationship remained when domain-general object shape recognition ability (*o*) was controlled for, suggesting that this relationship is specific to food recognition. However, it is possible that other general recognition abilities, such as those for color and texture information, could also play a role. To test this, we developed outdoor scene-recognition tests. Like images of prepared food, outdoor scenes are rich in color and texture information. In 204 participants, we replicated previous findings that food-recognition tasks remain correlated after controlling for* o*. We additionally found that these correlations remain when scene-recognition ability is controlled for, strengthening evidence for a food-specific visual ability. The negative relationship between food-recognition ability and food neophobia also persisted after controlling for both *o* and scene-recognition ability, supporting the idea that this relationship reflects food-specific processes. These results indicate that food-specific processing goes beyond general visual abilities that apply to shape, color, and texture. The relationship with food neophobia supports a connection between perceptual expertise for food and affective responses to food that could inform interventions for individuals with restricted eating patterns.

## Introduction

The measurement of individual differences in general visual object recognition abilities has emerged as a powerful tool for understanding specialized perceptual mechanisms. One framework centers on a domain-general ability for object recognition, called *o*, that accounts for shared variance across tasks that require discrimination among visually similar objects (Richler et al., 2019; Smithson & Gauthier, 2026). This ability generalizes across novel and familiar object categories and accounts for additional variance in visual performance, beyond general intelligence or other general cognitive abilities (Chow et al., 2025; Delavari et al., 2023; Smithson et al., 2024; Sunday et al., 2022). Measuring *o* facilitates the investigation of domain-specific visual abilities that are relevant to performance when solving specific tasks with objects from a specific category (e.g., matching fingerprints or identifying birds). For instance, we can ask if there is shared variance among different tasks from a specific domain, above and beyond the variance attributable to *o.* By controlling for the influence of *o,* we can isolate the domain-specific ability, which is typically related to domain-specific experience. For instance, when participants with musical experience were tested on different kinds of musical notation tasks, the tasks remained correlated even after controlling for *o* and other general abilities (Chang & Gauthier, 2021).

Recently, this approach has been extended to food recognition. Using two different tasks requiring discrimination among prepared food dishes, Gauthier and Fiestan (2023) found that while *o* accounted for a significant portion of the variance in performance on these tasks, there remained substantial shared variability between food-recognition tasks even after controlling for *o*. This food-recognition ability correlated negatively with food neophobia, which is the tendency to avoid novel foods (Rabadán & Bernabéu, 2021). A plausible interpretation of this correlation is that people with a history of seeking and experiencing novel foods are, as a result of these perceptual experiences, better at recognizing images of prepared food. This relationship survived when controlling for personality traits, general interest in food, and demographic variables, suggesting a specific relationship between visual food recognition abilities and food avoidance behavior. The relationship between perceptual abilities and food neophobia is particularly intriguing, given that food neophobia is a stable trait with important health implications, limiting dietary variety and predicting lower nutritional quality (Cole et al., 2017; Hazley et al., 2022).

Here, we revisit the claim for a food-specific ability, in acknowledgement of the need for stronger controls than were used in the original work. People can recognize images of prepared food based on multiple sources of information including shape, texture, and color. The measurement of *o* in Gauthier and Fiestan (2023) followed the established practice (Chang & Gauthier, 2021; Richler et al., 2019; Smithson et al., 2023; Smithson et al., 2024; Sunday et al., 2018) of using novel objects to avoid any variability in experience. The computer-generated novel objects used in these tasks vary in the shape of their parts and subtly differ in configuration – but while they are rendered in a color and a texture, these features do not vary among different objects. As such, color and texture are not diagnostic and controlling for *o* may not therefore capture all the domain-general individual differences in visual abilities that may be helpful for food recognition.

The ability to discriminate shapes is likely to be useful for food recognition judgments. Accordingly, *o* is correlated with food recognition performance (*r* = .40, Gauthier & Fiestan, 2023). Performance across different grayscale food-recognition tasks remains significantly correlated after controlling for *o*. This finding has been interpreted to suggest that some food-specific variance is independent of color (Sun & Gauthier, 2023). In addition, removing color from food-recognition tasks eliminated the relationship between food-recognition ability and food neophobia (Sun & Gauthier, 2023). One interpretation of this result is that the relationship between food-recognition ability and affective responses to food depends on food-specific color information. However, isolating a domain-specific mechanism requires careful consideration of all relevant domain-general abilities. It is unclear how much of the color-related processing of food images depends on domain-general color perception, or how much the ability to use texture information may be responsible for performance on the food-specific abilities in these studies.

While it is simple to remove color information from images, removing texture is more difficult. Here we take a different approach, using tasks with outdoor scenes to control for domain-general processes in food recognition that would not have been captured by *o*. Beyond color, texture information plays a crucial role in both food and scene perception. Both color and texture can help us distinguish between different types of meat, vegetables, or baked goods, and provide critical information about objects and surfaces in outdoor scenes, such as foliage, brick walls, or windows. Scenes can be successfully recognized at the “basic-level” (Rosch et al., 1976), applying labels like “forest,” “desert,” or “city” based on relatively low-level features like texture, color, and global orientation statistics, independently of object recognition (Greene & Oliva, 2009; Oliva & Torralba, 2006). Images of prepared food can be thought of as small-scale scenes whose recognition benefits from similar processes. The food-recognition ability measured by Gauthier and Fiestan (2023) is one that supports judgments at a relatively specific level of categorization – even though the tasks do not require naming the dish, the distractors are items that generally would have different names on a menu (e.g., “baklava” vs. “tiramisu” or “eggs florentine” vs. “eggs benedict”). Presented with different kinds of pasta dishes, observers are asked to judge which two are most similar, allowing for some differences in ingredients, preparation, and presentation.

The parallels between food and scene processing suggest that scene recognition tasks could provide a useful control for domain-general abilities that are not captured by traditional *o* measures. To this end, we developed new scene-recognition tests that mirror the format of the food-recognition tests. Like the food tests, these tests require participants to make fine discriminations between visually similar images based on their similarity. However, instead of judging which food dishes are the same or different, participants judge which outdoor scenes were taken from nearby or different locations. Performance on these scene tests should rely on the kind of shape processing measured by *o,* but also upon the processing of color and texture. The variance in test performance that is shared with food tests can be considered to reflect a domain general ability. By controlling for these additional domain-general visual abilities, we can provide a more stringent test of whether food recognition involves truly domain-specific mechanisms.

We also use these new scene-recognition tests to provide a stronger test of whether mechanisms that are truly food-specific support the relationship between food-recognition ability and food neophobia. If this relationship reflects food-specific mechanisms, rather than a general facility with processing color and texture information that could be associated with more general neophobia (Pliner & Hobden, 1992), it should persist even after controlling for color and texture perception ability.

This work has important implications for theoretical and applied questions in visual cognition and eating behavior. From a theoretical perspective, isolating food-specific visual mechanisms demonstrates that the human visual system can develop specialized processing for categories beyond those traditionally studied, like faces, words, or bodies (Mahon & Caramazza, 2011; Weiner et al., 2014). Identifying non-perceptual factors such as attitudes towards food and how they may relate to specific visual features, such as color, is important to understand what factors can drive category specialization in the brain (Jain et al., 2023; Pennock et al., 2023; Sun & Gauthier, 2023). The relationship between these visual mechanisms and food neophobia also raises applied questions about the development of perceptual expertise and eating behaviors. Food neophobia typically emerges during childhood and remains relatively stable throughout life. If specialized visual mechanisms for food recognition develop through experience, limited exposure to diverse foods during development could result in both increased food neophobia and reduced perceptual expertise. This could create a self-reinforcing cycle where poor recognition abilities increase the perceived novelty of foods, maintaining avoidance behaviors. Understanding these relationships could inform interventions for children and adults with restricted eating patterns.

## Method

### Participants

The correlation between food neophobia and food-specific recognition ability, after controlling for *o* in Gauthier and Fiestan (2023), was *r* = -.31. To detect an effect of this size with 90% power, we would need a sample size of 109. However, we sought a larger sample size for better precision of correlation estimates. Participants were tested online through the Prolific platform. We recruited only adult participants residing in the USA (18–45 years of age, although one reported being 46 years old on the day of testing), fluent in English, reporting normal or corrected-to-normal vision and no colorblindness, and with an approval rating on the platform of at least 95%. We collected data from 250 participants in August and September 2024, of which 234 completed all tasks. Those who failed at least one attention check were excluded from the dataset, resulting in a final sample size of 204 participants (108 identifying as women, 94 as men, one as “other,” and one preferring not to disclose their gender). The mean age of participants was 33.3 years (*SD* = 6.7). Ethics approval for the involvement of human subjects in this study was granted by the Vanderbilt University Institutional Review Board.

### Procedure

Participants were tested online through Prolific after providing affirmative consent. All participants were tested in a fixed order of tasks, including the order of trials within each task. This is a common practice in individual differences research, to prevent order effects from obscuring individual differences (Goodhew & Edwards, 2019), and in this work it was important that all participants completed the Food Neophobia Scale (FNS) prior to seeing food images. First, participants completed two measures of *o –* 3AFC Object Matching and Learning Exemplars. Next, they completed the FNS, followed by food matching, scene matching, food oddball, and scene oddball.

### Measures

#### Object recognition ability (o)

We estimated *o* using an approach validated in prior work (Smithson et al., 2024), which uses an aggregate score calculated from two different tasks, each using different novel object categories. Details of these tasks can be found in Smithson et al. (2024).

#### Three-alternative forced-choice (3AFC) matching – asymmetrical greebles

We employed a three-alternative forced-choice (3AFC) matching task to evaluate participants’ ability to match objects from a category of novel items (Asymmetrical Greebles). On each trial, following a fixation cross presented for 500 ms, participants were briefly shown a target Greeble for 300–1000 ms, followed by a category-specific mask for 500 ms. Next, three Greebles were displayed, and participants clicked on the Greeble that best matched the target. Participants had unlimited time to make their choice. The test included 51 trials, starting with a block of 11 trials featuring Greebles in the same orientation as the target. Subsequent trials included matches in different orientations from the target. Feedback was provided during the first three trials only. Performance was indexed by accuracy.

#### Learning exemplars

The Learning Exemplars Task required participants to study and recognize six target objects from a novel category (Vertical Ziggerins). In the initial study phase, the six target Ziggerins were presented on the screen for 20 seconds to familiarize participants with their features. This was followed by 6 trials of a 3AFC recognition test, where participants identified one studied Ziggerin from a set of three options presented on the screen. The other two options were unstudied Ziggerins. The six target Ziggerins were then shown again for an additional 20 seconds of study, followed by 18 3AFC trials. After a third study phase of 20 seconds, participants completed 24 more 3AFC trials, with Ziggerins presented from slightly different viewpoints, and some presented with Gaussian noise. Performance was indexed by accuracy.

#### Food neophobia scale (FNS)

We used the Food Neophobia Scale (FNS, Pliner & Hobden, 1992) to assess participants’ levels of food neophobia. Participants rated how much they agreed with each of ten statements (e.g., “how often do you try new food,” “I am afraid to eat things I have never had before,” “I will eat almost anything “) on a 7-point scale ranging from 1 (strongly disagree) to 7 (strongly agree).

#### Food-recognition ability

We measured food-recognition ability using the food-matching test and the food oddball test originally developed by Gauthier and Fiestan (2023). These tasks were designed to assess the ability to match different images of the same dish while incorporating distinct task demands. Both tasks targeted the category of “prepared food,” defined as dishes consisting of multiple ingredients that could be ordered from a menu or served as a single course at home. The tasks included a diverse range of visual experiences representative of what individuals in the USA encounter at home, in restaurants, or through media, with trials spanning a broad spectrum of difficulty. Easier trials involved distinctions such as differentiating between noodles and rice, while more challenging ones required identifying differences between similar dishes, such as flat versus round noodles. The food categories encompassed both typical American dishes and dishes from other cuisines, which ensures the test is sensitive to a range of perceptual abilities with food. Performance was indexed by accuracy.

#### Food matching

The food matching test included one example trial with the correct answer provided, followed by 60 test trials. On each trial, an image of prepared food was displayed for 1,000 ms, followed by a set of three images of prepared food. Participants were instructed to select the image that was “most similar to the studied image,” without time restriction. Only the first ten trials provided “correct/incorrect” feedback. The trials were designed to vary in difficulty: at the easiest level, the correct choice was a cropped version of the same image as the target food, while at the most difficult level, the correct choice was a completely different instance of the same dish, presented on different dishware or from a different perspective. Foils were carefully selected to share visual features with both the target food and its surrounding elements, such as the table or plate. Two additional trials were included as attention checks, featuring objects from different categories with similar shapes to ensure an obviously correct answer (e.g., tofu vs. Lego blocks). Performance was indexed by accuracy.

#### Food oddball

**Fig. 1:**
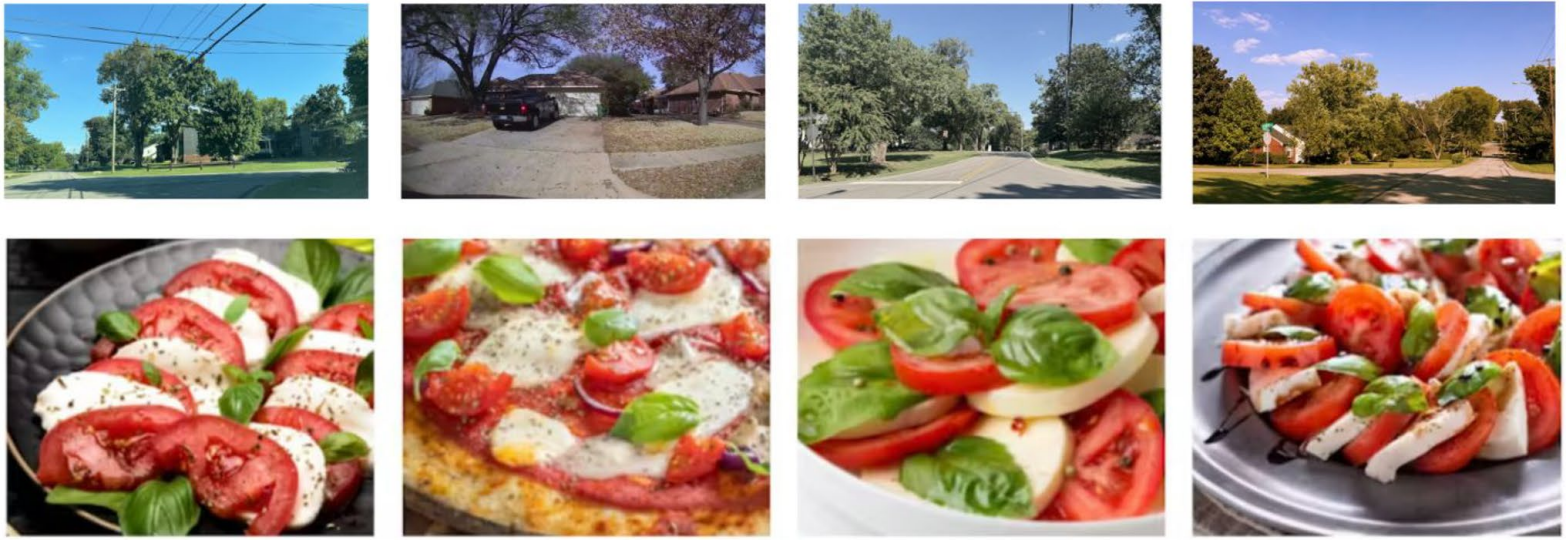
Example trials for food and scene oddball tasks. *Note.* Scene Oddball is top, Food Oddball is bottom, with the second image being the oddball in each case. Images are from copyright free photography (Food from Rouzes/E+, Carlo A/Moment, Yulia Naumenko/Moment, rudisill/E+ via Getty Images, Scenes from Isabel Gauthier) and are similar to those used in the test, which are copyrighted

The food oddball test used images of food collected in the same manner as the food-matching task but featuring different dishes. On each trial, four images of prepared food were presented: three depicted different versions of the same dish, while the fourth was a different dish, serving as the oddball (Fig. 1). Trial difficulty varied as a function of the oddball’s visual similarity to the other dishes. Participants were instructed to select the oddball on each trial without time restriction. The task included one practice trial followed by 60 test trials, with correct/incorrect feedback provided during the first five trials. Two additional trials were included as attention checks, where three of the images depicted different objects (e.g., dolls) instead of food. Performance was indexed by accuracy.

#### Scene-recognition ability

To measure scene-recognition ability, we created two tasks designed to assess participants’ capacities to make judgments about outdoor scenes from North America. For both the scene-matching test and the scene oddball test, we targeted generally urban areas, including small and large city scenes, containing some natural features but always including man-made features such as buildings, roads and/or vehicles.

Both tasks required judging which scenes were “from the same place.” All scenes were retrieved using Google Maps Panorama mode, with detailed location information recorded for each image, providing ground truth for the tasks. Trials were curated to vary in difficulty; easier trials involved scenes with distinct global features (e.g., more or less urban), while more challenging trials required distinguishing between subtle variations in similar environments (e.g., different neighborhoods of similar apparent population density). Foils were carefully selected to share visual characteristics, such as similar textures or colors, with the target images to enhance difficulty. License plate images were blurred, and plate information was not discernible. Text appearing in the scene stimuli was generally indiscernible. Textual information was not informative for these trials.

#### Scene matching

This test included one example trial in which the answer was provided, followed by 48 trials. On each trial, an image of an outdoor scene appeared for 2,000 ms, followed by a set of three images of outdoor scenes. Participants were asked to “click on the image that shows the scene which looks like it is from the same city/town as the scene you were originally shown,” without time restrictions. Only the first five trials included “correct/incorrect” feedback. Two trials were included as attention checks, with the foil options showing scenery images that were extremely different from the studied image (e.g., a snowy mountain view and a countryside scene). Performance was indexed by accuracy.

#### Scene oddball

This test used scenes collected in the same manner as in the scene-matching task, but from different locations. On each trial, four scenes were presented together: three depicted different areas or display angles of the same location, captured at the same or different times, and one was a different scene. These oddball scenes differed in visual similarity from the other scenes to varying extents, allowing for different trials to span a wide range of difficulty (Fig. 1). Participants selected the oddball on each trial, without any time restriction. There was one practice trial and there were 48 test trials, with the first five providing correct/incorrect feedback. Two attention-check trials were included in which one of the four images was a scene from an entirely different category, designed to stand out clearly from the other three (e.g., three images were identical views of a rural road, the fourth image showed an urban street scene).

#### Analyses

Aggregate scores were created for each construct by averaging across standardized scores for each of the relevant tests. These aggregates were then used as variables in multiple regression analyses. To test whether there was food-specific variance we predicted one food-recognition task with the other while controlling for demographic variables and other ability variables. To test whether there was a unique relationship between food recognition and food neophobia, we predicted food recognition with food neophobia, while also controlling for demographic variables, *o*, and scene recognition. To investigate the unique contributions of scene recognition and *o*, we compared models predicting food recognition that included both variables with models that included one or the other.

## Results

Descriptive statistics for each task are presented in Table 1.

**Table 1:** Descriptive statistics

Test	Mean (SD)	Min.	Max.	Skew	Kurtosis	Reliability
***O***						
3AFC matching greebles	71% (10%)	41%	94%	−0.33	−0.11	.70
Learn. Exemp. Ziggerins	56% (14%)	12%	88%	−0.10	−0.43	.82
Aggregate *o*	0 (0.78)	−2.23	2.06	−0.05	−0.18	.80
**Food recognition**						
Food matching	81% (9%)	50%	97%	−0.73	0.21	.78
Food oddball	63% (14%)	18%	88%	−0.74	0.89	.86
Aggregate food	0 (0.89)	−2.52	1.77	−0.44	−0.34	.89
**Scene recognition**						
Scene matching	63% (9%)	38%	83%	−0.27	−0.27	.59
Scene oddball	57% (13%)	19%	81%	−0.48	−0.34	.76
Aggregate scene	0 (0.86)	−2.65	1.74	−0.29	−0.35	.78
**Food neophobia**						
Food neophobia scale	29.5 (13.4)	10	68	0.70	0.02	.93

*Note.* Reliability estimates are Guttman’s ʎ_2_ (Callender & Osburn, 1979). Reliability estimates for aggregate scores were calculated in accordance with Wang and Stanley (1970)

### Zero-order correlations between tests

Table 2 reports zero-order correlations between visual tests. All these correlations were significant and positive (between.23 and.58). As expected, the two *o* tests show a more modest correlation than the pairs of scene or food tests, as they have neither the task nor the object category in common. These results are consistent with the idea that there is more in common between the food and scene tests (shape, texture, and color) than between each of these domains and the *o* tests (only shape).

**Table 2:** Zero-order Pearson correlations between visual tests

	1.	2.	3.	4.	5.
1. Food matching	-				
2. Food oddball	.579	-			
3. Scene matching	.457	.425	-		
4. Scene oddball	.489	.540	.470	-	
5. 3AFC matching	.282	.281	.286	.311	-
6. Learning exemplars	.383	.281	.406	.386	.228

*Note.* All correlations *p* <.01. All correlations remain significant when Bonferroni correction for family-wise error rate is applied

### Zero-order correlations between aggregate scores and food neophobia

We created equal-weight aggregate scores for food recognition, scene recognition, and *o.* All three aggregates were positively correlated (Table 3). Food neophobia was negatively correlated with food recognition, while it was not significantly correlated with *o*, replicating Gauthier and Fiestan (2023). There was a small and nonsignificant negative correlation between food neophobia and scene recognition, consistent in direction with the idea that food neophobia could correlate with food recognition partly for domain-general reasons, but also consistent with the view that domain-general factors cannot explain the relationship.

**Table 3:** Zero-order correlations between visual abilities and food neophobia

	1. Food	2. Scene	3. *o*
1. Food recognition	-		
2. Scene recognition	.627 ***	-	
3. *o*	.440 ***	.517 ***	-
4. Food neophobia	-.226 **	-.133	-.078

*Note.* * *p* <.05, ** *p* <.01, *** *p* <.001. All significant correlations remain significant when a Bonferroni correction for family-wise error rate is applied

### Multiple regression analyses

Multiple regression analyses were conducted to ask whether there was still evidence of food-specific variability when controlling for domain-general abilities. Following the logic in Gauthier and Fiestan (2023), we predicted scores on one food-recognition task (Food Oddball) with the other food-recognition task (Food Matching). Food Matching was a significant predictor of Food Oddball, when controlling for age and gender, as well as *o* and scene recognition (Table 4). Similar results were obtained when we switched the two food tasks and predicted Food Matching using Food Oddball as a predictor (Table 5). These results provide evidence for a food-specific ability.

**Table 4:** Multiple regression predicting Food Oddball

	Est.	*SE*	*t*	*p*
Age	-.058	0.055	−1.052	.294
Gender	-.297	0.107	−2.770	.006
*o*	.020	0.063	0.318	.751
Scene recognition	.360	0.070	5.179	<.001
Food matching	.361	0.066	5.480	<.001

*Note.* Gender is coded as women = 0, not women = 1. Beta coefficients are standardized except for gender. *F*(5, 198) = 31.69, *p* <.001; Adj*. R*^2^ =.431

**Table 5:** Multiple regression predicting Food Matching

	Est.	*SE*	*t*	*p*
Age	.092	0.055	1.671	.096
Gender	-.105	0.110	−0.953	.342
*o*	.16	0.063	2.561	.011
Scene recognition	.239	0.073	3.289	.001
Food oddball	.365	0.067	5.480	<.001

*Note.* Gender is coded as women = 0, not women = 1. Beta coefficients are standardized except for gender. *F*(5, 198) = 30.92, *p* <.001; Adj. *R*^2^ =.424

**Fig. 2:**
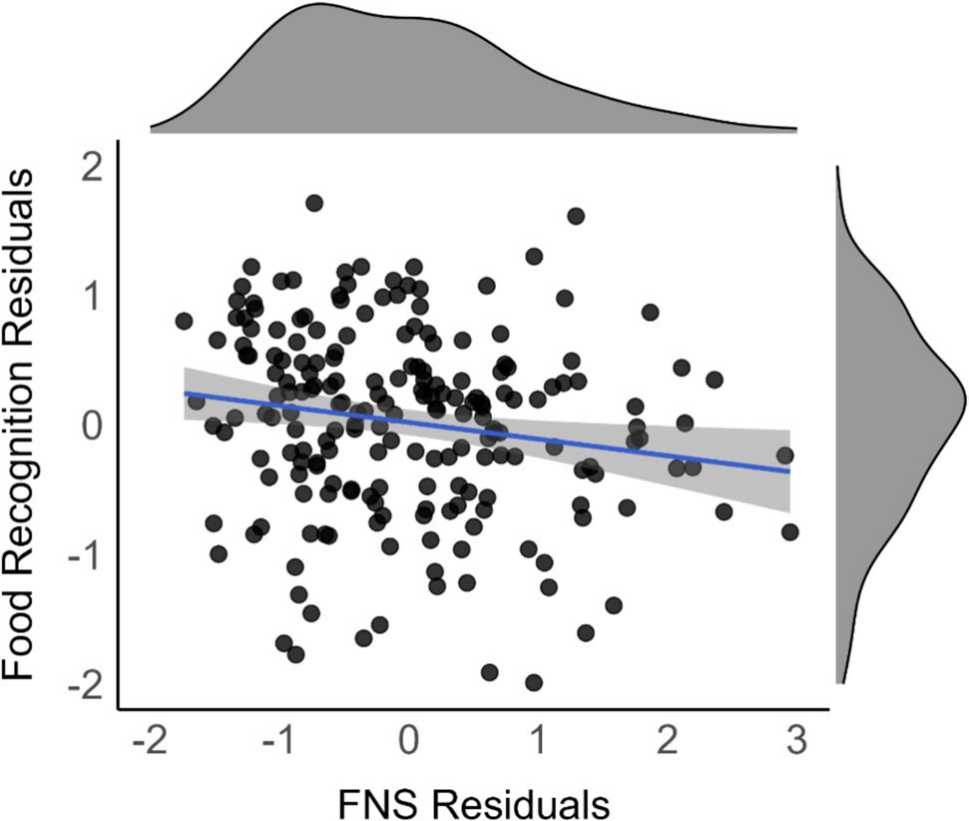
Partial correlation between Food Neophobia and Food Recognition. *R*_*partial*_ = -.158, 95% CI [-.029, -.021], *R*^*2*^ =.025, *p* =.024, controlling for age, gender, *o*, and scene recognition

Another multiple regression tested whether food neophobia predicted food-recognition ability, when controlling for age, gender, *o*, and scene recognition (Table 6). Replicating Gauthier and Fiestan (2023), women^1^ had better food-recognition abilities than men, whereas age was not a significant predictor. Serving as the critical test of our main hypothesis, food neophobia was a significant predictor of food recognition, even after controlling for scene recognition (Fig. 2).

**Table 6:** Multiple regression predicting Food Ability

	Est.	*SE*	*t*	*p*
Age	.032	0.054	0.595	.553
Gender	-.321	0.106	−3.029	.003
*o*	.16	0.061	2.581	.011
Scene recognition	.51	0.063	8.139	<.001
Food neophobia scale	-.12	0.053	−2.257	.025

*Note.* Gender is coded as women = 0, not women = 1. Beta coefficients are standardized except for gender. *F*(5, 198) = 33.54, *p* <.001; Adj. *R*^2^ =.445

To assess the relative contributions of *o* and scene recognition, we compared models predicting food recognition that did not include *o* or scene recognition with those that included either *o* or scene recognition or both. The model including only age, gender, and food neophobia as predictors explained 9.9% of the variance (adjusted *R*^2^). The model with *o* added as a predictor explained 26.3% of the variance, explaining an additional 16.4% of total variance (*F*(1) = 45.494, *p* <.001). The model with scene recognition, but not *o*, added as a predictor explained 42.9% of the variance, explaining an additional 33% of total variance (*F*(1) = 116.67, *p* <.001). When *o* was added as a predictor to this model, the total variance explained only rose slightly to 44.5%. This suggests that scene recognition captures most of the variance in food recognition that can be explained by *o*. This result could be expected as scenes include objects. However, *o* still accounted for a small but statistically significant unique portion of the variance when both predictors were included (Table 6), and overall model fit was significantly improved by *o*’s addition (*F*(1) = 6.660, *p* =.011).

## Discussion

We sought to test the strength and specificity of individual differences in food-recognition ability by controlling for a broader range of domain-general visual abilities than in previous work. We replicated findings of significant shared variance between food-recognition tasks when controlling for *o*, and we extended this result by showing that these correlations persist when additionally controlling for performance on scene proximity judgment tasks that rely heavily on color and texture processing. Importantly, the relationship between food-recognition ability and food neophobia remained significant after accounting for both *o* and scene-recognition ability. Together, these results provide stronger evidence for a food-specific visual ability that is distinct from general object recognition and scene-recognition ability.

These findings reinforce the interpretation that food-recognition ability, as measured by the tests created by Gauthier and Fiestan (2023), relies on domain-specific mechanisms. As expected, *o* is related to judgments with scenes and food, both of which include objects. When predicting food recognition, *o* adds a small but significant increase in variance explained beyond that offered by scene recognition, possibly because shape recognition is better measured by *o* tests than by scene-recognition tests. This could be because objects in scenes are not as similar (and therefore challenging) as novel objects, or because the additional spatial, texture, and color information in scenes makes shape information less critical. While we cannot say for certain which properties of the food and street scene stimuli participants rely on to make their judgments, stimuli from both categories vary in color and texture, whereas these properties are held constant across the novel objects used on any given trial in the *o* tasks. These conjectures need to be tested in further studies that include additional measures for all constructs, thereby increasing both reliability and construct coverage.

The survival of food-specific correlations between food-recognition tests and between food recognition and food neophobia when *o* and scene recognition are controlled for points to the presence of visual mechanisms that are specialized for recognizing prepared foods. These may develop through visual experience with food, as we expect similar mechanisms could do for any other category with which people have a great deal of experience. Theoretically, these findings contribute to the understanding of domain specificity in visual cognition. While extensive work has characterized specialized processing for faces, bodies, and words (e.g., Peelen & Downing, 2005; White & Burton, 2022; Wong & Gauthier, 2007), our results suggest the visual system may also support specialized recognition of prepared food. This is consistent with neuroimaging research finding food-specific activity in the visual system (Jain et al., 2023; Khosla et al., 2022; Pennock et al., 2023). Together, the behavioral and neural signs of specialization warrant further study of which aspects of our experience with food may support specialization (Ritchie et al., 2024).

The relationship between food-recognition ability and food neophobia may be an example of how visual perceptual expertise can interact with affective responses. Individuals who avoid novel foods may have fewer opportunities to develop rich visual representations of a variety of prepared dishes, potentially limiting their perceptual discrimination ability. This relationship between food recognition and food neophobia appears to be supported by color, as it disappears when food-recognition ability is tested with grayscale images (Sun & Gauthier, 2023; see also Mueller & Gauthier, 2025). Color likely plays an important role in the development of affective responses to food, as men who are colorblind have lower levels of food neophobia than those who have normal color vision (Gauthier & Olatunji, 2024; Sun & Gauthier, 2023). Richer and more diverse color-based visual exposure may support the development of perceptual expertise, while reinforcing familiarity and positive associations with food. Individuals with reduced exposure to visually varied foods, whether due to behavioral avoidance or perceptual limitations like colorblindness, may encode fewer distinct representations of prepared dishes, leading to diminished recognition ability and heightened neophobic responses. The interplay between visual perceptual mechanisms and affective traits like food neophobia may thus reflect a developmental feedback loop, where reduced exposure leads to weaker recognition, increasing perceived novelty, and reducing motivation to explore new foods. Understanding this perceptual-affective coupling could inform strategies aimed at reducing food neophobia, such as limiting chromatic information while increasing visual exposure to diverse foods.

Beyond their use as a control variable, the scene-recognition tasks offer a new tool for assessing domain-general visual abilities. Most of the work in scene perception involves categorization (e.g., is this a forest, a city, or a beach?) or spatial navigation (Bar, 2004; Boone et al., 2019; Burte et al., 2018; Epstein & Baker, 2019; Oliva & Torralba, 2006). The ability to make proximity judgments is likely related to both kinds of judgments, although they do not require precise spatial decisions and they depend on finer distinctions than most categorization tasks. We chose proximity judgments because they are difficult perceptual judgments like those required in the food tasks, but in a domain so clearly different that any shared variance with food recognition would be attributable to domain-general processes. The scene tests could also prove useful in studying spatial navigation, environmental perception, or memory for real-world settings. It would also be interesting to explore the domain-specific aspects of the ability to judge location proximity from images, for instance, whether it is related to measures of spatial navigation ability (Boone et al., 2019; Burte et al., 2018).

### Limitations

Several limitations should be acknowledged. While our scene tasks approximate the perceptual demands of the food tasks more closely than prior controls, they may not fully capture all sources of domain-general variance relevant to food recognition. Our main goal was to measure variability relevant to texture and color, but there are likely other sources of diagnostic information that are shared and unshared between scenes and food that could be systematically investigated. The correlation between food-recognition ability and food neophobia is relatively small (although it replicates across studies, Gauthier & Fiestan, 2023; Mueller & Gauthier, 2025; Sun & Gauthier, 2023) and it could reflect unspecified third variables. We did not use a latent variable approach (Bollen, 2002) as has been used in some of our other work on visual abilities (Richler et al., 2019; Smithson et al., 2025; Sunday et al., 2018). This approach would have several advantages, including estimation of inter-construct relationships that are free of measurement error, which may be larger, and the ability to partition task variance into construct-relevant and construct-irrelevant variance. Future larger-scale research could use a greater number of tasks to allow for such a latent-variable modeling approach to be successful. Additionally, there is the possibility of order effects due to fixed task order. Our present results could motivate future work into the structure of domain-general visual abilities, separating the contributions of features like shape, texture, and color.

## Footnotes

1 To use all participants, we coded gender as women vs. non-women, with only two participants included in the non-women group who did not report a gender or reported “other.”
